# Application Value of A Clinical Radiomic Nomogram for Identifying Diabetic Nephropathy and Nondiabetic Renal Disease

**DOI:** 10.2174/0115734056332507250210105723

**Published:** 2025-02-24

**Authors:** Xiaoling Liu, Weihan Xiao, Jing Qiao, Xiachuan Qin

**Affiliations:** 1 Department of Ultrasound, Beijing Anzhen Nanchong Hospital, Capital Medical University (Nanchong Central Hospital), Nanchong, Sichuan, China; 2 Department of Ultrasound, Chengdu Second People’s Hospital, Chengdu, Sichuan 610000, China; 3 North Sichuan Medical College, Nanchong, Sichuan Province, China; 4 Department of Obstetrics and Gynecology Ultrasound, Affiliated Hospital of North Sichuan Medical College, Nanchong, Sichuan 637000, China

**Keywords:** Chronic disease, Diabetic nephropathy, Ultrasonography, Machine learning

## Abstract

**Objective:**

An ultrasound-based radiomics Machine Learning Model (ML) was utilized to assess non-invasively the conditions of diabetic nephropathy and non-diabetic renal disease in diabetic patients.

**Methods:**

A retrospective examination was conducted on 166 diabetic patients who had undergone renal biopsies guided by ultrasound, with the group comprising 114 individuals diagnosed with DN and 52 NDRD. The participants were randomly divided into the training set and the testing set (7:3). Following the extraction of radiomics features from the renal ultrasound images, a univariate analysis was conducted, and the Least Absolute Shrinkage And Selection Operator (LASSO) algorithm was applied to select the most significant features. Three ML algorithms were applied to construct the prediction models. Subsequently, the patients' clinical characteristics were evaluated through both univariate and multivariate logistic regression analyses, which facilitated the development of a clinical model, following a clinical radiomics model was formulated, integrating the radiomics scores (Radscore), along with the independent clinical variables identified through the screening process. The diagnostic performance of the three models constructed was evaluated using the receiver operating characteristic (ROC) curve analysis.

**Results:**

Among the three radiomics ML models, the logistic regression (LR) model achieved the best performance, with the area under the curve (AUC) values of 0.872 (95%CI, 0.800-0.944) and 0.836 (95%CI, 0.716-0.957) for the training set and the testing set, respectively. The decision curve analysis (DCA) verified the clinical practicability of the ML model. Within the same testing set, the AUC of the clinical model was 0.761 (95%CI, 0.606-0.916). The nomogram model based on clinical features plus Radscore showed the best discrimination, with an AUC value of 0.881 (95%CI, 0.779-0.982), which was better than that of the single clinical model and the radiomics model.

**Conclusion:**

The ML model of radiomics based on ultrasound images has potential value in the non-invasive differential diagnosis of patients with diabetic nephropathy. The nomogram constructed based on rad score and clinical features could effectively distinguish DN from NDRD.

## 1 INTRODUCTION

The global incidence of diabetes is increasing every year. Diabetic Nephropathy (DN) ranks among the most prevalent and severe complications associated with diabetes [1], and it is recognized as a primary contributor to the development of end-stage renal disease [2]. Compared with Nondiabetic renal disease (NDRD), DN has a relatively poor renal prognosis [3, 4]. The treatment and prognosis of NDRD differ from those of DN due to differences in pathological type [5-8]. In NDRD, a greater emphasis is placed on the treatment of primary conditions, most of which are curative or may be alleviated with the use of glucocorticoids and immunosuppressants. Therefore, accurately distinguishing between DN and NDRD in diabetic patients is important for effectively guiding clinical treatment. However, currently, the clinical diagnosis of DN is based mainly on clinical manifestations. In the absence of renal biopsy, misdiagnosis may occur [9, 10]. Some studies showed that when diabetes patients receive renal biopsy, NDRD accounts for 36%~52% [11-14]. Many international guidelines and expert consensus [15, 16], such as the Kidney Disease Quality of Life Guidelines (KDOQI) guidelines, agreed that the possibility of NDRD should be considered for many atypical symptoms such as without diabetes retinopathy, rapid decline of GFR, rapid increase of proteinuria, and intractable hypertension. At present, it is generally believed that renal biopsy should be actively carried out in patients with diabetes with suspected non-diabetic kidney disease to confirm the pathological diagnosis. Renal biopsy is currently the only gold standard diagnostic method for evaluating diabetic kidneys, although invasive renal puncture is limited by several contraindications in clinical practice. Moreover, patients are also fear of renal puncture, which causes several diabetic patients to opt out of undergoing the puncture operation even if clinical symptoms exist [17]. However, certain scholars have attempted to distinguish DN from NDRD based on several clinical indicators, such as diabetic retinopathy, hypertension, duration of diabetes, HbA1c, and urinary RBC [18-20], with accuracy, sensitivity, and specificity ranging from approximately 68~72%, 68~86%, and 66~72%, the results of such attempts remain much lower than the clinically required standards.

Radiomics allows for the extraction and quantification of high-throughput imaging biomarkers beyond human perception [21-23]. The use of these biomarkers in combination with various Machine Learning (ML) techniques in previous studies by our research group confirmed that the renal ultrasound-based radiomics model could effectively monitor the subtle conditions of chronic kidney disease [24, 25]. However, to the best of the author’s knowledge, no study has reported noninvasive assessments of DN and NDRD statuses using ultrasound-based radiomics. Therefore, in the present study, the radiomics features of renal US were extracted to establish an ML model that could noninvasively evaluate the DN and non-NDRD status. Furthermore, clinical indicators and radiomics scores (Radscores) were determined, and a nomogram was established to guide treatment in clinical settings.

## 2 METHODS

### 2.1 Study Design and Population

The medical records of patients who underwent renal biopsies at two hospitals from January 2018 to May 2023 were retrospectively reviewed. The inclusion criteria were as follows: 1. complete clinical data and ultrasound (US) images; and 2. more than 10 glomeruli were visible under a light microscope. The exclusion criteria were as follows: 1. renal artery stenosis or urinary tract obstruction; 2. patients who underwent renal transplantation, had renal tumours, or had renal failure; 3. severe heart, lung, and liver dysfunction, coagulation abnormalities, or severe infection; 4. poor-quality ultrasound images that could not be used for image analysis; or 5. DN combined with NDRD. The participants were randomly assigned into training and testing groups in a 7:3 ratio. The screening process is illustrated in Fig. (**1**).

The following clinical parameters were collected: sex, age, weight, systolic blood pressure (SBP), diastolic blood pressure (DBP), C-reactive protein (CRP), urine red blood cell (URBC/µL), serum uric acid (SUa), glycosylated haemoglobin (HbA1c), total cholesterol (TCHO), high-density lipoprotein cholesterol (HDL-C), low-density lipoprotein cholesterol (LDL-C), estimated glomerular filtration rate (eGFR), the total volume of urine (TVU), and 24-h urine protein excretion (TUPE), renal length, renal width, cortical thickness, main renal artery flow velocity (MRA), segmental renal artery (SRA).

### 2.2 Ultrasound image Acquisition, Renal Biopsy, and Pathology

GE Vivid E9, E20 (GE Healthcare, USA), EPIQ5 (Philips, Netherlands), and Resona 7 (Mindray, China) were used. In addition, convex array probes with a range of frequencies from 2 to 6 MHz (models C5–2 and V6–2) were utilized. All patients were examined while lying in the supine position after overnight fasting. The ultrasound transducer was carefully positioned on the upper right quadrant of the abdomen at an angle, and the patient was asked to suspend breathing momentarily as the ultrasound image capturing the largest cross-sectional view of the right kidney was obtained. The long diameter of the kidney was selected as the longitudinal section for capturing the image. All examinations were performed during apnoea at the end of the inspiratory phase.

Two experienced nephrologists performed percutaneous renal biopsy of the right kidney within 3 days after renal ultrasound examination. The biopsy specimens were evaluated for pathological diagnosis using immunofluorescence, optical, or electron microscopy. Both renal biopsy and pathological diagnosis were performed according to the guidelines of the Kidney Disease Outcomes Quality Initiative (KDOQI) [26] of the National Kidney Foundation. The pathological analysis was independently completed by two pathologists with more than 8 years of experience.

### 2.3 Construction of the Clinical Model

The measurement data were subjected to a significance test followed by univariate logistic regression analysis to examine the relationship between the clinical indicators and the occurrence of the presence or absence of DN. The independent variables with statistical significance (*P* < 0.05) were selected and included in univariate and multivariate logistic regression analysis to determine the independent predictors that were significantly associated with the presence of DN or NDRD for the establishment of the clinical model.

### 2.4 Construction of the Radiomics Model

**Fig. (2):**
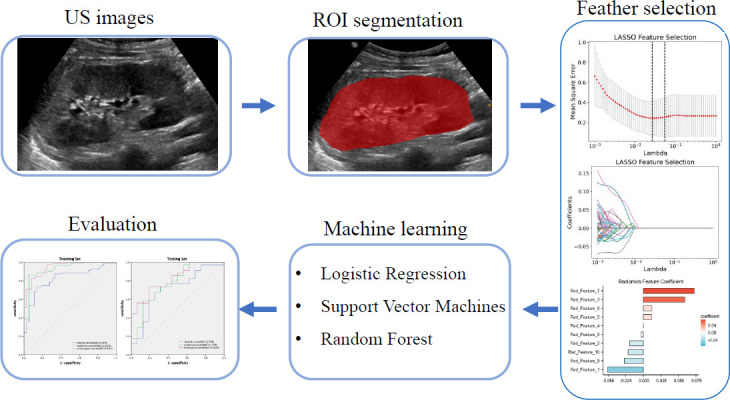
The process of establishing ultrasound-based radiomics models.

The Region Of Interest (ROI) was manually selected and segmented by Reader 1 (who had 10 years of renal ultrasound experience) and Reader 2 (with 12 years of experience in renal ultrasound) using ITK-SNAP (version 3.8.0, http://www.itksnap.org/pmwiki/pmwiki.php?n=Downloads.SNAP3). The PyRadiomics package in Python software (version v3.0.1, https://github.com/AIM-Harvard/pyradiomics) was then applied to extract features from the ultrasound images (Fig. **2**). The extracted features included the original image, wavelet, square, square root, logarithm, index, gradient, local binary pattern (LBP) 2D, and LBP 3D filters. Two weeks later, the renal ultrasound images of 50 patients were selected randomly and resegmented first by the same radiologist and then by another radiologist who had 12 years of experience in ultrasound imaging to determine the intraclass correlation coefficient (ICC). The features with an ICC < 0.75 were excluded and were not used in the subsequent analysis.

First, the data were divided randomly into a training set (n = 116) and a testing set (n = 50) at a ratio of 7:3. The data in the training set were used for training the prediction model. In contrast, the data in the testing set were used for evaluating the performance of the model. In the training set, the radiomics features of the DN set and the NDRD set were compared, and the radiomics features that conformed to a normal distribution were subjected to an independent sample t-test. Images that did not conform to a normal distribution were subjected to the Mann‒Whitney U test. The features with significant differences were screened out. Next, the optimal lambda parameters were adjusted, followed by least absolute shrinkage and selection operator (LASSO) regression analysis to determine the most significant features to be used for predicting DN through 5-fold cross-validation.

Furthermore, three radiomics models were constructed, and their Radscores were calculated using various ML algorithms, namely, logistic regression (LR), random forest (RF), and support vector machine (SVM). The selected features were used for training the models. The training set was utilized to conduct a five-fold cross-validation process to identify the optimal parameter settings. The parameters for the three ML algorithms were fine-tuned using the grid search technique coupled with a fivefold cross-validation (CV) approach within the training set. In each cycle of CV, the hyperparameters that yielded the highest area under the curve (AUC) of receiver operating characteristic (ROC) were selected, and the complete training set was employed to finalize the model [27, 28]. The testing set was then used to evaluate the performance of the established models. After each round of CV, the prediction probability was assigned to each patient.

### 2.5 Statistical Analysis

Statistical analysis was performed using IBM SPSS Statistics 20.0 (IBM Corp., Armonk, NY, USA) and Python 2.2 (Python Software Foundation, Beaverton, OR, USA). Normally distributed quantitative data are expressed as the mean ± standard deviation. The quantitative data with a nonnormal distribution are expressed as the median ± interquartile range. Categorical data are expressed as numbers and percentages. Comparative analysis was conducted using the chi-square test and the independent sample t-test. Statistical significance was determined by bilateral *P* values that were less than 0.05 (**Supplementary Material**).

## 3 RESULTS

### 3.1 Clinical Characteristics of the Patients

According to the renal biopsy and pathology results, the 166 patients could be divided into a DN cohort (114 patients) and an NDRD cohort (52 patients). In this group, there are 159 patients with type 2 diabetes, 6 patients with type 1 diabetes, and 1 with steroid diabetes. The pathological types of NDRD included 13 cases of membranous nephropathy, 28 cases of IgA nephropathy, and 1 case of mesangial proliferative glomerulonephritis. The 166 patients had an average age of 50.92 ±10.09 years. Among the 116 patients, 106 patients were included in the training set, while the remaining 50 patients were included in the testing set. The clinical characteristics of the patients are presented in Table **1**.

### 3.2 Clinical Model

According to the single factor analysis of the count data and the significance analysis of the results of the independent sample t-test conducted for the measurement data, it was concluded that the differences in the parameters of age (*P* = 0.003), SBP (*P* = 0.026), URBC (*P* = 0.008), HbA1C (*P* = 0.01), TCHO (*P* = 0.037), LDL (*P* = 0.038), and eGFR (*P*<0.001) were statistically significant between the DN and NDRD set. The above factors were, therefore, included in the subsequent univariate and multivariate regression analysis, which revealed URBC (OR 0.994; 95% CI: 0.984–1.005, *P* = 0.016) and eGFR (OR 0.970; 95% CI: 0.948–0.993, *P* = 0.001) as the independent predictors of DN and NDRD. The ROC of the clinical model training set based on URBC and eGFR was 0.777, while that of the testing set was 0.761.

### 3.3 Radiomics Model

A total of 836 radiomics features were extracted from the ultrasound images. The intragroup and intergroup correlation analyses and univariate correlation analysis revealed a total of 220 features that were significantly different between the DN and NDRD sets. LASSO and multivariate logistic regression analyses of these significant factors identified the 10 most significant features, which were subsequently used for constructing the ultrasonic radiomics model. The features used for model construction were as follows: original_gldm_Small DependenceLowGrayLevelEmphasis, original_ngtdm_Stren- gth, wavelet-LHH_glrlm_HighGrayLevelRunEmphasis, wave let-LHH_glrlm_LowGrayLevelRunEmphasis, wavelet-HLH_ firstorder_Mean, wavelet-HLH_glrlm_RunLengthNonUnifor mity, wavelet-HHL_firstorder_ Maximum, wavelet-HHL glszm_ SmallAreaLowGrayLevelEmphasis, wavelet-HHH_ glszm_ZonePercentage, and wavelet-LLL_gldm_SmallDepen denceHighGrayLevelEmphasis.

**Fig. (4):**
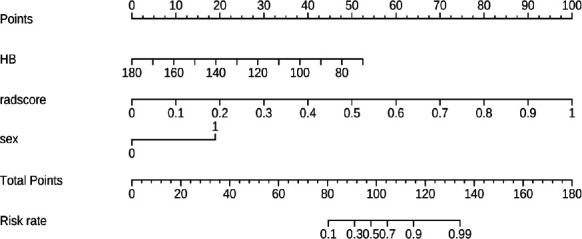
The decision curve for distinguishing DN and NDRD among three ml models in the training (**A**) and testing sets (**B**).

**Fig. (5):**
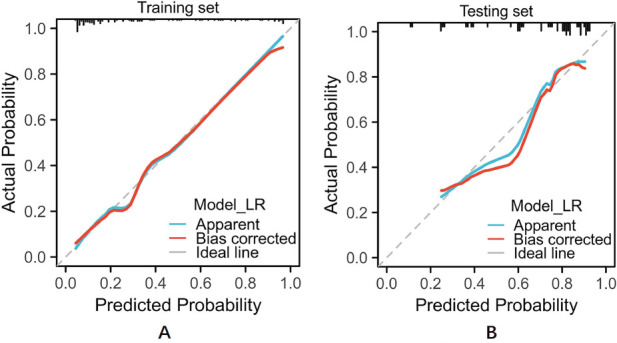
The calibration curve for the clinical radiomics model LR. (**A**) The calibration plot indicated a close match between the predicted probabilities for the training set; (**B**) The calibration plot reflected a strong concordance with the probabilities in the testing set.

The diagnostic performance of the three radiomics models based on the different ML algorithms is presented in Table **2**. The ROC curves of these models in the training and testing sets are depicted in Fig (**3**). Among the three constructed models, the LR model demonstrated the most favorable performance. The AUC, accuracy, sensitivity, specificity, positive predictive, and negative predictive values of this model were 0.872 (95%CI, 0.800-0.944), 83.62%, 88.75%, 72.22%, 87.65% and 74.29%, respectively, in the training set and 0.836 (95%CI 0.716-0.957), 82.0%, 82.49%, 81.28%, 90.31% and 68.42%, respectively, in the testing set. The decision curve and calibration curve analysis verified the clinical practicability of the LR model (Figs. **4** and **5**).

### 3.4 Clinical Radiomics Nomogram

**Fig. (6):**
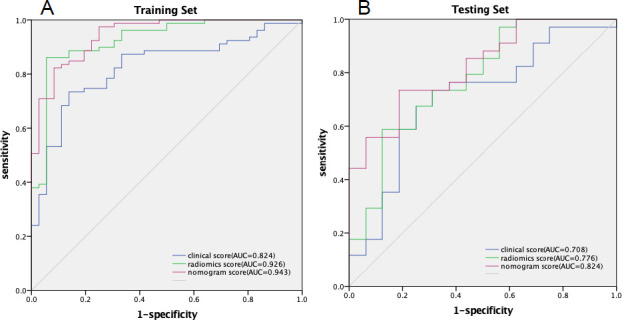
The nomogram of the clinical radiomics model. **A**: The values of the clinical characteristics and the Rad scores were converted into quantitative values according to the points axis. **B**: After summing the individual points to achieve the final sum, presented on the total points axis, the evaluation of DN/NDRD was obtained.

A clinical radiomics nomogram was established by combining the Radscore and clinical features (Fig. **6**). The AUC, accuracy, sensitivity, and specificity of the combined nomogram model were 0.894 (95%CI, 0.831-0.958), 87.1%, 91.3%, and 77.8%, respectively, in the training set and 0.881(95%CI, 0.779-0.982), 86.0%, 91.2%, and 75.0%, respectively, in the testing set.

As shown in Table **3** and Fig. (**7**), the nomogram model based on the clinical indicators and Radscore was significantly better than the single radiomics model and the clinical model.

## 4 DISCUSSION

The accurate diagnosis of diabetic nephropathy is important for effectively guiding clinical treatment plans. In the present study, features were extracted from the renal ultrasound images of diabetic patients, and the extracted features were subsequently screened to obtain the 10 most significant radiomic features. These 10 features were then used for constructing three ML models that could enable the differentiation of DN and NDRD in diabetic patients. Among the models that were constructed, the LR model achieved the best diagnostic efficiency, with AUC values of 0.872 and 0.836 in the training set and the testing set, respectively. Furthermore, a nomogram model was constructed by combining the independent clinical factors with the Radscore. The AUC of this model in the testing set was 0881, which was significantly better than that of the single ultrasound-based radiomics model (0.836) and the clinical model (0.761). To the best of the authors’ knowledge, this model is the first nomogram model based on both clinical indicators and the US Radscore for the noninvasive diagnosis of diabetic nephropathy.

Ultrasound (US) is a clinically convenient, noninvasive, and green detection method that is suitable for the diagnosis of kidney diseases. However, US examination enables the observation of only the gross structure of the kidney [29]. Radiomics, on the other hand, allows for the transformation of traditional imaging data into high-dimensional data that may be mined, thus enabling the characteristics of lesions that cannot be distinguished by the naked eye to be obtained [30]. Certain scholars have attempted to explore the correlation between the quantitative parameters of contrast-enhanced ultrasound and the severity of renal injury in DN patients. Accordingly, it was concluded that contrast-enhanced ultrasound could suitably reflect changes in glomerular lesions [31]. In recent years, radiomics analysis via renal US has been applied to study chronic kidney disease. Bandara, MS [32], and colleagues employed radiomic features derived from US images to detect chronic kidney disease in kidneys. They showed that radiomic features, which are based on wavelet transform, are responsive to the directional properties of US speckle patterns and can effectively differentiate patients with chronic kidney disease from those who are healthy. In a study by our research group [24, 25], an ML model for predicting the activity of patients with lupus nephropathy was established by extracting radiomics features from renal US images. The ML model achieved the best results in the testing group, with an AUC of 0.822. Considering the great potential of ultrasound-based radiomics in the diagnosis of nephropathy, in the present study, the two-dimensional ultrasound features of the kidney and the high-dimensional ultrasound imaging features were extracted noninvasively to construct three ML models for distinguishing DN and NDRD in diabetic patients. The LR model exhibited the best performance, with an area under the ROC curve of 0.872 in the training set and 0.836 in the testing set, higher than the clinical model (including the conventional ultrasound feature) with 0.777 in the training set and 0.761 in the testing set. Therefore, the ML model established by extracting radiological features from renal ultrasound images could effectively distinguish between DN and NDRD. The results also demonstrated that ultrasound-based radiomics model images have great potential in the identification and prediction of DN and NDRD.

The results of the univariate and multivariate analyses revealed that the URBC and eGFR were independent predictors of DN and NDRD. Owing to the differences in the natural course of the two diseases, the changes in the eGFR in DN patients from a high filtration state in the early stage of the disease to a decline in the later stage also differ^33^. Moreover, numerous large cohort studies conducted in recent years have revealed that a simple decrease in the eGFR is not uncommon in the diabetic population^34^. Therefore, eGFR alone cannot be used for suitably predicting and reflecting the state of DN. In addition, the URBC values were significantly different between the DN and NDRD sets in the present study. This was attributed to the relatively high proportion of IgA nephropathy in the NDRD, and haematuria is often considered one of the characteristics of IgA nephropathy. Haematuria was revealed as an independent predictor in the present study, consistent with the findings reported by Hsieh, J. T.^18^. According to the URBC and eGFR values, a clinical prediction model was established in the present study. However, the ROC curve achieved using this model did not fulfill the clinical requirements. Therefore, further improvement of the diagnostic efficiency was attempted by combining clinically independent factors with the Radscore to generate a radiomics nomogram model. In contrast, the clinical model underscored the significance of fundamental clinical data in the non-invasive assessment of DN and NDRD, and the radiomics model that leverages ultrasound images encompasses the process of image quantification. The clinical radiomics model amalgamates the strengths of both clinical and radiomics approaches, thereby enhancing the model's overall predictive performance.

As with all research, the current research also encountered specific limitations. First, the study's retrospective nature made it susceptible to potential selection bias. The retrospective design of the study was prone to selection bias. There is inevitably a certain selection bias in this set of data. For example, in most cases of severe proteinuria and mild renal function damage, in order to avoid exacerbating the deterioration of renal function in patients with severe renal function damage, clinical physicians may choose to temporarily suspend puncture biopsy and receive symptomatic treatment for a certain period. In subsequent research, we will include multiple clinical data examination results and timeline variables such as disease duration to reduce such errors. Second, the sample size of the study was small. Therefore, future studies must be conducted with larger sample sizes to allow for the generalization of the results to performance verification. Finally, it is recommended that the integration of multimodal ultrasound could potentially enhance the precision of the developed model, representing a prospective research avenue for our research group [33, 34].

## CONCLUSION

In summary, a clinical radiomics nomogram model based on clinical and ultrasound radiomics features was established. The model exhibited high accuracy in distinguishing between DN and NDRD in diabetic patients. The nomogram can provide clinicians with further personalized imaging information to noninvasively assess the status of DN and NDRD in diabetic patients, thereby guiding the selection of treatment strategies.

## Figures and Tables

**Fig. (1):**
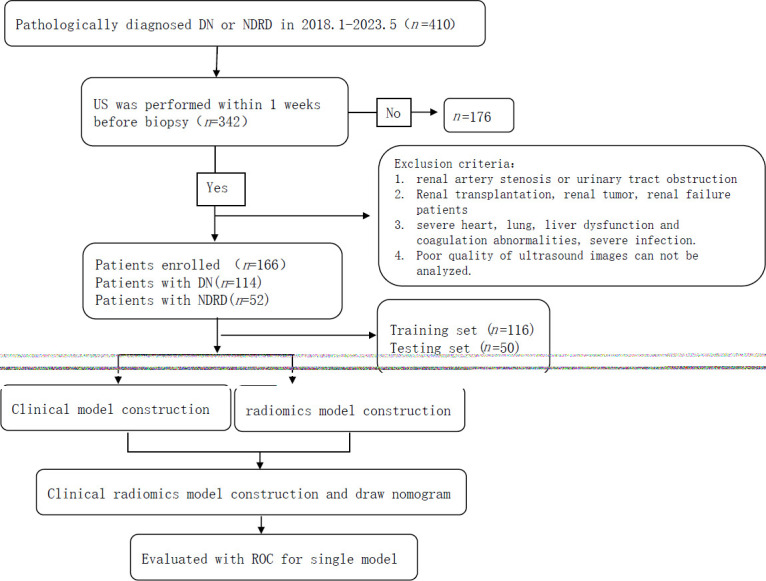
Flowchart of the research process.

**Fig. (3):**
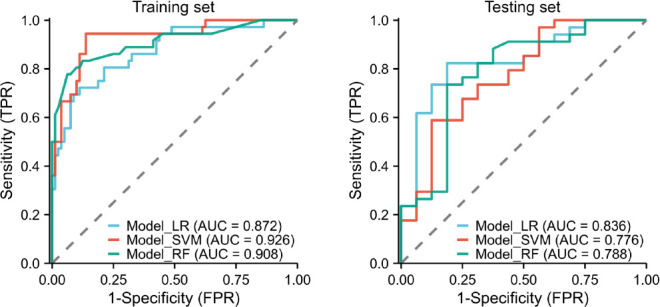
The ROC curves of the three ML models in the training and testing set.

**Fig. (7):**
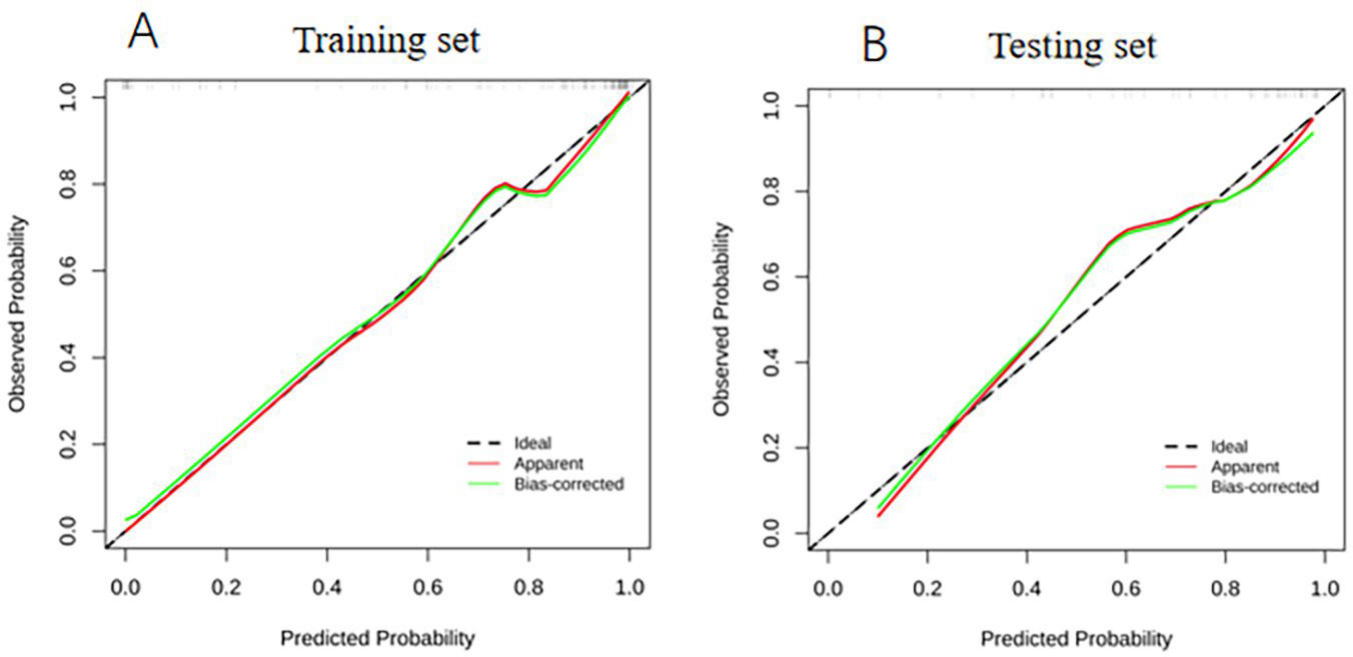
The ROC curves of the clinical model, radiomics model and clinical radiomics model. The clinical radiomics model showed certain advantages in both the training (**A**) and testing (**B**) set.

**Table 1:** The basic clinical characteristics of the training set and the testing set.

**Clinical factor**	**DN**	**NDRD**	***P***
Sex (male/female)	76/38	33/19	0.678
Age(y)	52.39 ± 10.41	47.67 ± 11.94	0.011
Weight(kg)	71.26 ± 11.63	67.25 ± 14.12	0.056
SBP(mmHg)	147.77 ± 22.21	140.35 ± 16.15	0.016
DBP(mmHg)	90.10 ± 12.19	88.92 ± 14.54	0.151
CRP (mg/L)	5.65 ± 12.29	11.22 ± 24.26	0.051
URBC (/μL)	19.58 ± 41.65	48.98 ± 78.44	0.002
HbA1C (%)	7.46 ± 1.59	7.04 ± 1.30	0.049
eGFR(mL/min/1.73m2)	58.41 ± 27.89	76.46 ± 20.31	0.000
TCHO (mmol/L)	5.18± 1.89	5.83 ± 2.05	0.044
LDL (mmol/L)	3.12 ± 1.34	3.64 ± 1.57	0.029
HDL (mmol/L)	1.17± 0.37	1.31 ± 0.53	0.059
TVU(L/24h)	1.77 ± 0.56	1.61 ± 0.48	0.070
UPE(g/24h)	2.19 ± 2.36	2.61 ± 2.67	0.301
Sua (μmol/L)	386.57 ± 101.95	379.61 ± 108.20	0.690
Renal length	108.62 ± 10.58	106.79 ± 8.35	0.272
Renal width	51.37± 5.59	49.19± 7.09	0.055
Cortical thickness	7.70 ± 1.41	7.61 ± 1.43	0.691
MRA	69.91± 13.73	75.07± 16.37	0.036
SRA	42.85± 13.40	43.44± 8.95	0.772

**Abbreviations: SBP**, systolic blood pressure; DBP, diastolic blood pressure; CRP, C-reactive protein; URBC, urine red blood cell; SUa, serum uric acid; HbA1c, glycosylated hemoglobin; TCHO, total cholesterol; HDL-C, high-density lipoprotein cholesterol; LDL-C, low-density lipoprotein cholesterol; EGFR, epidermal growth factor receptor; TVU, total volume of urine; TUPE, 24-h urine protein excretio. SRA, segmental renal artery; MRA, main renal artery flow velocity.

**Table 2:** Performance of the three ultrasound-based radiomics models in the training and testing set.

**Model**	**AUC (95% CI)**	**SEN**	**SPE**	**PPV**	**NPV**	**ACC**
**Training set**						
**LR**	0.872 (0.800-0.944)	88.75%	72.22%	87.65%	74.29%	83.62%
**SVM**	0.926 (0.827-0.980)	86.25%	94.44%	97.18%	75.56%	88.79%
**RF**	0.908 (0.843-0.976)	96.25%	77.78%	90.59%	90.32%	90.52%
**Testing set**						
**LR**	0.836 (0.716-0.957)	82.49%	81.28%	90.31%	68.42%	82.0%
**SVM**	0.776 (0.631-0.921)	67.65%	75.36%	85.19%	52.17%	70.39%
**RF**	0.788 (0.639-0.937)	82.35%	68.75%	84.85%	64.71%	78.43%

**Abbreviations: LR**, logistic regression; SVM, support vector machine; RF, random forest; AUC, area under the curve; CI, confidence interval; SEN, Sensitivity; SPE, Specificity; PPV, positive predictive value; NPV, negative predictive value, ACC, Accuracy.

**Table 3:** Performance of the conventional ultrasound model, clinical model, radiomics model and clinical radiomics model in the training and testing set.

**Model**	**AUC (95% CI)**	**ACC**	**SEN**	**SPE**	**PPV**	**NPV**
**Training set**						
**Clinical**	0.777 (0.692-0.861)	70.7%	66.3%	80.6%	88.3%	51.8%
**Radiomics**	0.872 (0.800-0.944)	83.6%	88.8%	72.2%	87.7%	74.3%
**Clinical Radiomics**	0.894 (0.831-0.958)	87.1%	91.3%	77.8%	90.1%	80.0%
**Testing set**						
**Clinical**	0.761 (0.606-0.916)	76.0%	73.5%	81.2%	89.3%	59.1%
**Radiomics**	0.836 (0.717-0.956)	82.0%	82.4%	81.2%	90.3%	68.4%
**Clinical Radiomics**	0.881(0.779-0.982)	86.0%	91.2%	75.0%	88.6%	80.0%

**Abbreviations: AUC**, area under the curve; CI, confidence interval;ACC, Accuracy, SEN, Sensitivity; SPE, Specificity; PPV, positive predictive value; NPV, negative predictive value.

## Data Availability

The data that support the findings of this study are available on request from the corresponding author, [XC.Q]. The data are not publicly available due to their containing information that could compromise the privacy of research participants.

## LIST OF ABBREVIATIONS

ML: Machine Learning Model
ROC: Receiver operating characteristic
LR: Logistic regression
DCA: Decision curve analysis
AUC: Area under the curve
DN: Diabetic Nephropathy
NDRD: Nondiabetic renal disease
KDOQI: Kidney Disease Quality of Life Guidelines
SBP: Systolic blood pressure
DBP: Diastolic blood pressure
CRP: C-reactive protein
URBC/µL: Urine red blood cell
SUa: Serum uric acid
HbA1c: Glycosylated haemoglobin
TCHO: Total cholesterol
HDL-C: High-density lipoprotein cholesterol
LDL-C: Low-density lipoprotein cholesterol
eGFR: Estimated glomerular filtration rate
TVU: Total volume of urine
MRA: Main renal artery flow velocity
SRA: Segmental renal artery
ROI: Region Of Interest
LBP: Local binary pattern
ICC: Intraclass correlation coefficient

## STANDARDS OF REPORTING

STROBE guidelines were followed.
