# Supplementary material for: Application Value of A Clinical Radiomic Nomogram for Identifying Diabetic Nephropathy and Nondiabetic Renal Disease
Source: Curr Med Imaging. 2025 Feb 24;21:e15734056332507. doi: 10.2174/0115734056332507250210105723 (PMC13176756; doi:10.2174/0115734056332507250210105723)
Supplement: Supplementary file 1 — Supplementary material is available on the publisher's website along with the published article. [file CMIM-21-E15734056332507_SD1.pdf]

1573-4056/25 *Send Orders for Reprints to reprints@benthamscience.net*

BENTHAM SCIENCE

CrossMark

Current Medical Imaging

Content list available at: https://benthamscience.com/journals/cmir

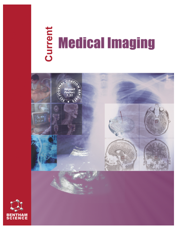

Supplementary Material

Application Value of A Clinical Radiomic Nomogram for Identifying Diabetic Nephropathy and Nondiabetic Renal Disease

Xiaoling Liu[1,#], Weihan Xiao[3,#], Jing Qiao[4,#] and Xiachuan Qin[2,*]

[1]*Department of Ultrasound, Beijing Anzhen Nanchong Hospital, Capital Medical University (Nanchong Central Hospital), Nanchong, Sichuan, China*
[2]*Department of Ultrasound, Chengdu Second People's Hospital, Chengdu, Sichuan 610000, China*
[3]*North Sichuan Medical College, Nanchong, Sichuan Province, China*
[4]*Department of Obstetrics and Gynecology Ultrasound, Affiliated Hospital of North Sichuan Medical College, Nanchong, Sichuan 637000, China*

```
model_params = {
# -------------- Logistic Regression ------------------
'LR': {
'model': LogisticRegression(random_state=60),
'params': {
'C': np.logspace(-3, 3, num=7)
}
},
# -------------- SVM-rbf ------------------
'SVM': {
'model': svm.SVC(probability=True, random_state=60),
'params': {
'kernel': ['rbf', 'linear'],
'C': np.logspace(-2, 3, num=6),
'gamma': np.linspace(0.01, 100, num=10)
}
},
# -------------- Random Forest ------------------
'RF': {
'model': RandomForestClassifier(n_jobs=-1, random_state=60),
'params': {
'n_estimators': np.arange(10, 1001, 10),
'max_depth': np.arange(1, 11),
'max_features': np.arange(1, 11)
}
},
}
```

© 2025 The Author(s). Published by Bentham Science Publisher.

This is an open access article distributed under the terms of the Creative Commons Attribution 4.0 International Public License (CC-BY 4.0), a copy of which is available at: https://creativecommons.org/licenses/by/4.0/legalcode. This license permits unrestricted use, distribution, and reproduction in any medium, provided the original author and source are credited.

DOI: 10.2174/0115734056332507250210105723, 2025, *21*, e15734056332507
